# miR-452-5p suppressed the metastasis of Non-small cell lung cancer through regulating Moesin

**DOI:** 10.7150/jca.83221

**Published:** 2023-07-03

**Authors:** Jing Zhuang, Jianjun Fan, Lihuan Zhu, Lilan Zhao, Yangyun Huang, Xiaojie Pan, Tianxing Guo

**Affiliations:** 1 Department of Thoracic Surgery, Shengli Clinical Medical College of Fujian Medical University, Fujian Provincial Hospital, No. 134 East Street, Fuzhou 350001, China.; 2 Department of Plastic and Burn, Shengli Clinical Medical College of Fujian Medical University, Fujian Provincial Hospital, No. 134 East Street, Fuzhou 350001, China.

**Keywords:** miR-452-5p, NSCLC, MAPK, ERK

## Abstract

**Background**: Non-small cell lung cancer (NSCLC) is a common malignant tumor, and it is characterized by high mortality. MicroRNA-452-5p (miR-452-5p) and Moesin (MSN) have been proved to be related with regulation of tumors. If miR-452-5p could regulate NSCLC through targeting MSN remain unclear.

**Methods**: TargetScan data and GEPIA databases were used to predict binding site and analyze gene expression, respectively. EdU staining, wound healing, and Transwell assays were performed to measure cell proliferation, migration, and invasion, respectively.

**Results**: The binding site between miR-452-5p and MSN was predicted and validated. Overexpression of miR-452-5p cell lines were constructed, and miR-452-5p mimics markedly inhibited the migration, invasion, and proliferation ability of both H322 and A549 cells, but these effects of miR-452-5p were reversed by pcDNA-MSN. pcDNA-MSN significantly reversed the influence of miR-452-5p mimics on the EMT related proteins expression in H322 and A549 cell lines by decreasing E-cadherin and increasing N-cadherin. Significant higher expression of MSN in lung adenocarcinoma and lung squamous cell carcinoma was observed through GEPIA and TCGA data base analysis. Higher expression of MSN is positively correlated with advanced lung cancer and suggests poor prognosis.

**Conclusions**: We demonstrated that miR-452-5p modulated the cell proliferation, migration, invasion, and EMT process of H322 and A549 cell lines through targeting MSN. This research might provide a novel prevention and treatment target for NSCLC.

## 1. Introduction

Lung cancer is a common malignant tumor. Its incidence and mortality are increasing recently, which seriously threatens people's life safety 1. It is difficult to detect lung cancer in early stage in time. When diagnosed, it is almost in the advanced stage, and the prognosis is very poor 2. According to its pathological classification, lung cancer can be divided into non-small cell lung cancer (NSCLC) and small cell lung cancer (SCLC), and NSCLC accounts for 80% of lung cancer 3.

The traditional treatment of lung cancer is chemotherapy. However, drug resistance will gradually develop in the course of treatment and eventually lead to treatment failure 4. The side effects of chemotherapy drugs often cause fatal effects. With the research on the biological behavior and targeted drugs of NSCLC, molecular targeted therapy has made significant progress 5. However, the targeted drug resistance rate for these targets is high and the applicable population is limited 6. It is of great clinical significance to actively carry out the research on the pathogenesis of lung cancer and to find the corresponding tumor treatment targets.

E-cadherin is the most critical intercellular adhesion molecule. E-cadherin makes adjacent cells establish adhesive connections, helps epithelial cells assemble into epithelial slices, inhibits cell movement, and maintains the resting state of cells in epithelial slices 7. The expression of E-cadherin is significantly down-regulated and its function is partially or completely lost in both early and advanced malignant tumors 8. The process of promoting these changes is known as epithelial-mesenchymal transition (EMT) 9. Therefore, in-depth understanding of the molecular mechanism of lung cancer invasion and metastasis will help to reveal the essence of lung cancer occurrence and development, and provide an effective way to treat lung cancer invasion and metastasis.

MicroRNA (miRNA) is a kind of cellular endogenous non-coding RNA molecule with the function of regulating the translation of protein, which is about 22 nucleotides long 10, 11. It was reported that miR-452-5p exerted different effects in different types of tumors 12, 13. The regulatory role of miR-452-5p in NSCLC remains unclear, and if miR-452-5p could affect the EMT process of NSCLC cells has not been reported.

Moesin (MSN) is a member of the Ezein, root protein and membrane protein family, and is a connecting molecule between the membrane and cytoskeleton of epithelial cells 14, 15. However, if miR-452-5p could regulate NSCLC through affecting MSN has not been reported.

In this study, bioinformatics methods were performed to analyze the relationship between MSN expression and prognosis. Overexpression of MSN and miR-452-5p in H322 and A549 cell lines were constructed. The influence of MSN and miR-452-5p on the cell proliferation, migration, invasion, and apoptosis were measured. We firstly demonstrated miR-452-5p regulated the cell proliferation, migration, invasion, and EMT process of H322 and A549 cell lines through targeting MSN. This study might provide a new understanding for the regulatory role of miR-452-5p in NSCLC.

## 2. Materials and methods

### 2.1 Cell culture

H1703, H1299, A549, H460, H322, and HNBE cell lines purchased from Chinese Academy of Science (Beijing, China) were used in this research. Cells were cultured with DMEM medium (gibco, #12491015, Langley, OK, USA) containing 5% FBS, 30 μg/ml streptomycin, and 50 IU penicillin at 5% CO_2_ and 37℃.

### 2.2 EdU staining

EdU solution (1:1000, ab219801, Abcam, UK) was added to cells for incubation (24 h) at 37 ℃ with 5% CO2. Then, polyformaldehyde was used to fix cells (15 min). After washing with PBS (5 min/time, 3 times), the cells incubated with DAPI dye for 2 min. Finally, cells were analyzed with a fluorescence microscope.

### 2.3 Wound healing

When cells grown to 70% confluence, they were scraped with a 200 μL pipette. the distance between wound was tested at 0 h and 48 h, respectively. Finaly, the migration distance was analyzed.

### 2.4 RT-PCR

RNA was extracted with the TRIzol agent (#15596026, Invitrogen, USA). Reverse transcriptase (TAKARA) were used to synthesize the complementary DNA. qRT-PCR was performed with SYBR green qPCR Mix kit (Genstar). The primers of are listed as follows: miR-452-5p (F: GCGCAACTGTTTGCAGAG, R: GTGCAGGGTCCGAGGT), MSN (F: GATGCTGTCCTGGAATATCTGA, R: TCTGCTCATAGATGTTGAGACC), U6 (F: CTCGCTTCGGCAGCACA, R: AACGCTTCACGAATTTGCGT).

### 2.5 Western blotting

The proteins were measured with BCA method. SDS-PAGE was conducted, and protein samples were transferred to a PVDF membrane (Milipore, GVWP02500, US). The membrane was blocked with non-fat milk, and washed with TBST. Then, the membranes were incubated with primary antibodies and second antibodies, successively. Finally, enhanced chemiluminescence detection kit (Thermo Fisher, USA) was used in immunoblots. Rabbit monoclonal to Moesin (1:1000, ab169789, Abcam, UK), rabbit monoclonal to N-Cadherin (1:1000, ab76011), rabbit monoclonal to E-Cadherin (1:2000, ab212059), rabbit polyclonal to beta-actin (1:3000, ab8227).

### 2.6 Transwell assay

Cells (2×10^5^) were seeded in the upper chamber with a matrix gel (1:6 diluted, Corning) with FBS-free medium. The lower chamber was added with DMEM containing 5% FBS. After 24 h, the invasive cells in the lower chamber were fixed with 70% ethanol, and stained with crystal violet staining. Finally, the invasive cells were analyzed.

### 2.7 Binding site prediction and validation

TargetScan data was used to predict binding site between MSN and miR-452-5p, and luciferase reporter assay was conducted to validate this binding site. Briefly, wild-type and mutant reporters (MSN-WT and MSN-MUT), and NC mimics and miR-452-5p mimics were co-transfected in cells with Lipofectamine 3000. Luciferase activity was measured after 24 h. The relative luciferase activity was detected by dual-luciferase reporter assay in cells. The mRNA level of miR-452-5p was measured with RT-PCR method. The mutation sequences were listed as follows: MSN mut (F: TCTACATCTTTATGTACTCTACTGATA), MSN mut (R: ACATAAAGATGTAGAAAGAAGAAGAGC).

### 2.8 Bioinformatics analysis

GEPIA (http://gepia.cancer-pku.cn/) and TCGA (https://www.cancer.gov/about-nci/organization/ccg/research/structural-genomics/tcga) and were used to analyze gene expression and survival rate.

### 2.9 Cell transfection

miR-452-5p mimics, pcDNA-MSN, and related controls were designed and obtained from Realgene (Shanghai, China). Plasmids transfection was performed with lipofectamine 2000 (#11668019, Invitrogen, US) according to the instruction. Briefly, cells in logarithmic phase were seeded in 24-well plate, and transfection was conducted. pcDNA 3.1/v5-his-topo plasmid (Thermo Fisher Scientific, Waltham, USA) without cDNA was used as the empty vector group, and cells without transfection was used as the blank group. After Geneticin (#10131035, Thermo Fisher, US) screening, stable transfection was achieved.

### 2.10 Statistical analysis

Results were statistically analyzed using SPSS (Version 20). Significance was set at p-value < 0.05. T-test and ANOVA test was used for p value determination.

## 3. Results

### 3.1 The mRNA expression level of miR-452-5p in NSCLC tissue and lung cancer cell lines was significantly suppressed

**Figure 1:**
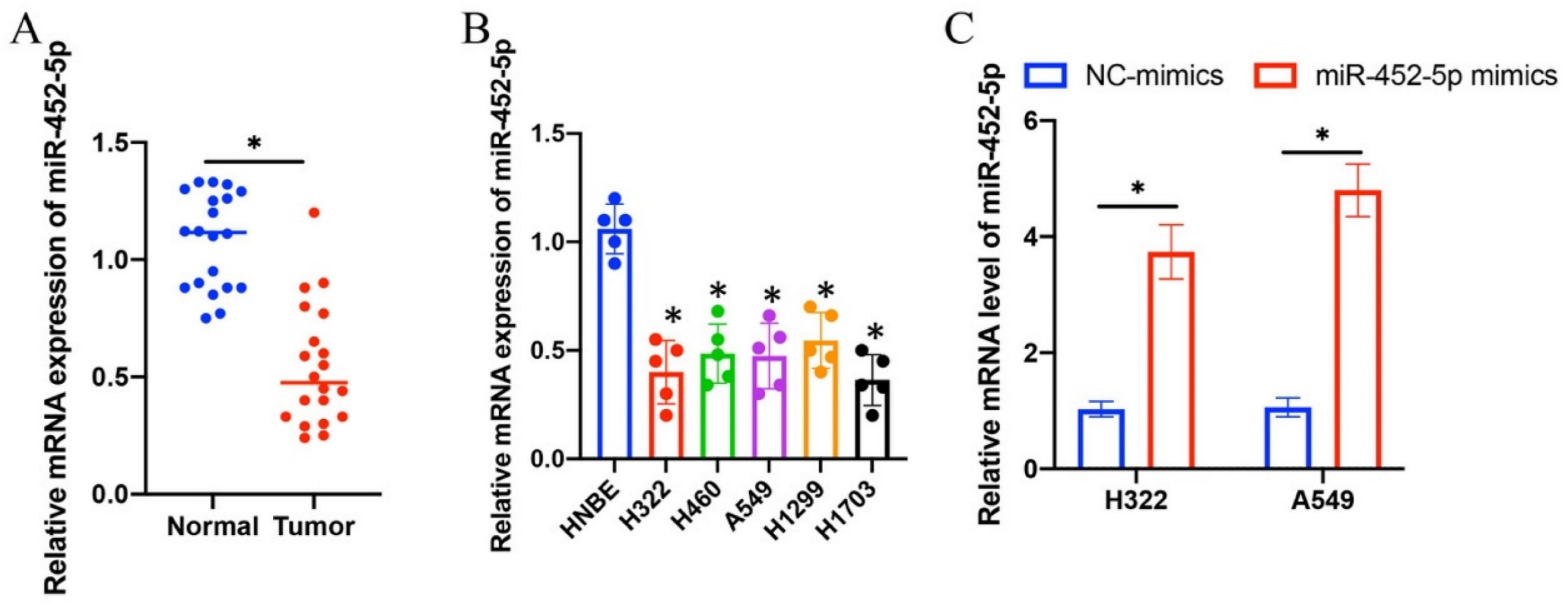
**The mRNA expression level of miR-452-5p in NSCLC tissue and lung cancer cell lines was significantly suppressed*.*** (A) The mRNA expression of miR-452-5p in NSCLC tissues was significantly restrained compared with group normal; (B) The mRNA expression of miR-452-5p in H322, H1299, H1703, A549, and H460 cell lines was significantly inhibited compared with group HNBE cell line; (C) Overexpression vectors of miR-452-5p were constructed and transfected successfully in H322 and A549 cell lines. * suggests p<0.05.

The expression intensity of miR-452-5p in NSCLC tissues and lung cancer cell lines (H322, H1299, H1703, A549, and H460) was markedly suppressed compared with normal lung tissues and HNBE cell line, respectively (Figure 1 A-B). The overexpression vectors of miR-452-5p were constructed and transfected successfully in H322 and A549 cell lines (Figure 1 C).

### 3.2 miR-452-5p mimics markedly inhibited the migration, invasion, and proliferation ability of both H322 and A549 cells

**Figure 2:**
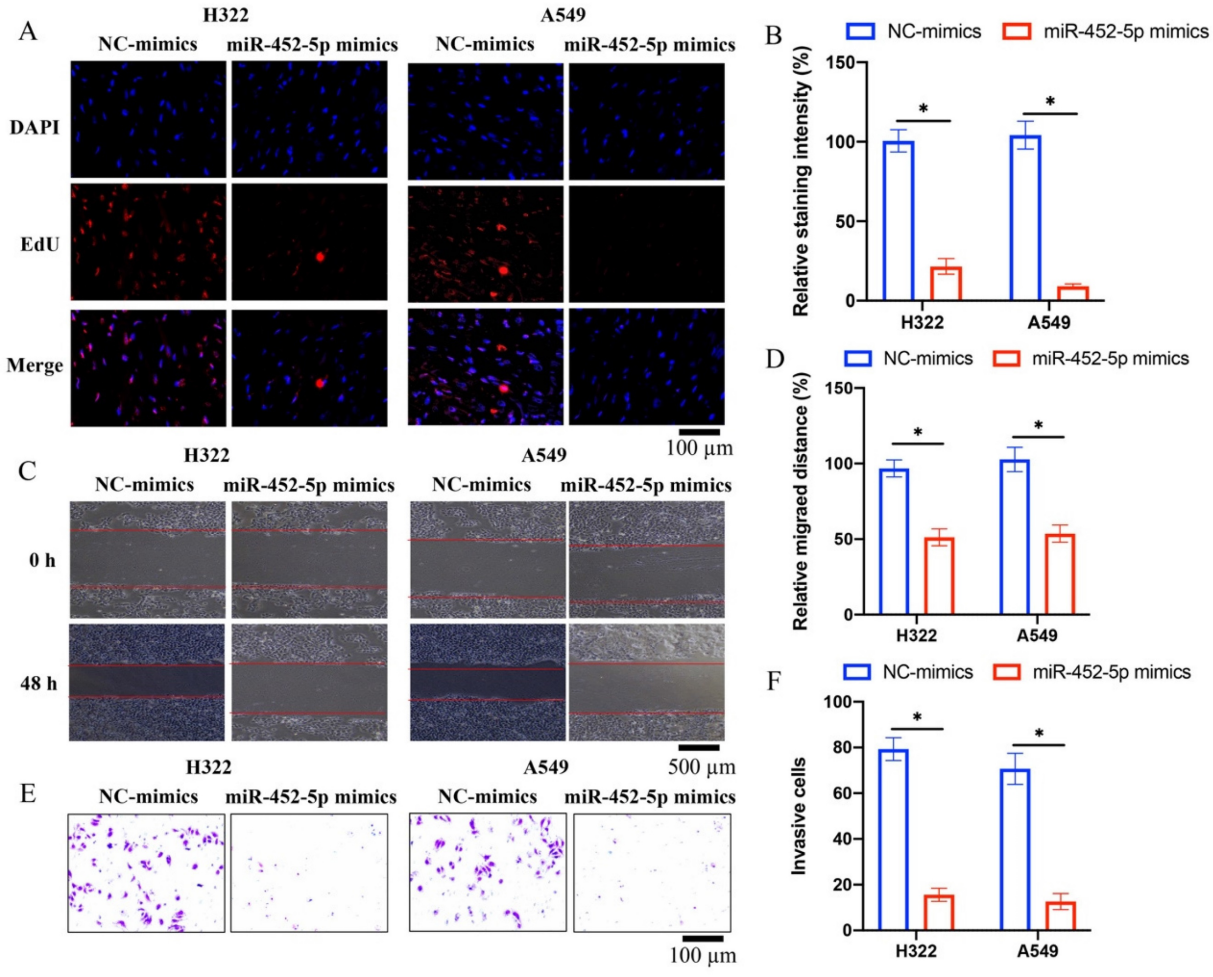
**miR-452-5p mimics markedly inhibited the migration, invasion, and proliferation ability of both H322 and A549 cells*.*** (A) The cell proliferation was evaluated with EdU staining method; (B) miR-452-5p greatly suppressed the proliferation of A549 and H322 cells; (C) The cell migration was measured with wound healing assay; (D) miR-452-5p greatly inhibited the migration of A549 and H322 cells; (E) The cell invasion was measured with Transwell assay; (F) miR-452-5p greatly inhibited the invasion of A549 and H322 cells. * suggests p <0.05.

Overexpression of miR-452-5p in A549 and H322 cell lines was successfully established, and the invasion, migration, and proliferation ability of A549 and H322 cell lines were evaluated. We found that miR-452-5p could greatly suppress the ability of proliferation (Figure 2 A-B), migration (Figure 2 C-D), and invasion (Figure 2 E-F) of A549 and H322 cells compared with group control.

### 3.3 The binding site between miR-452-5p and MSN was validated

**Figure 3:**
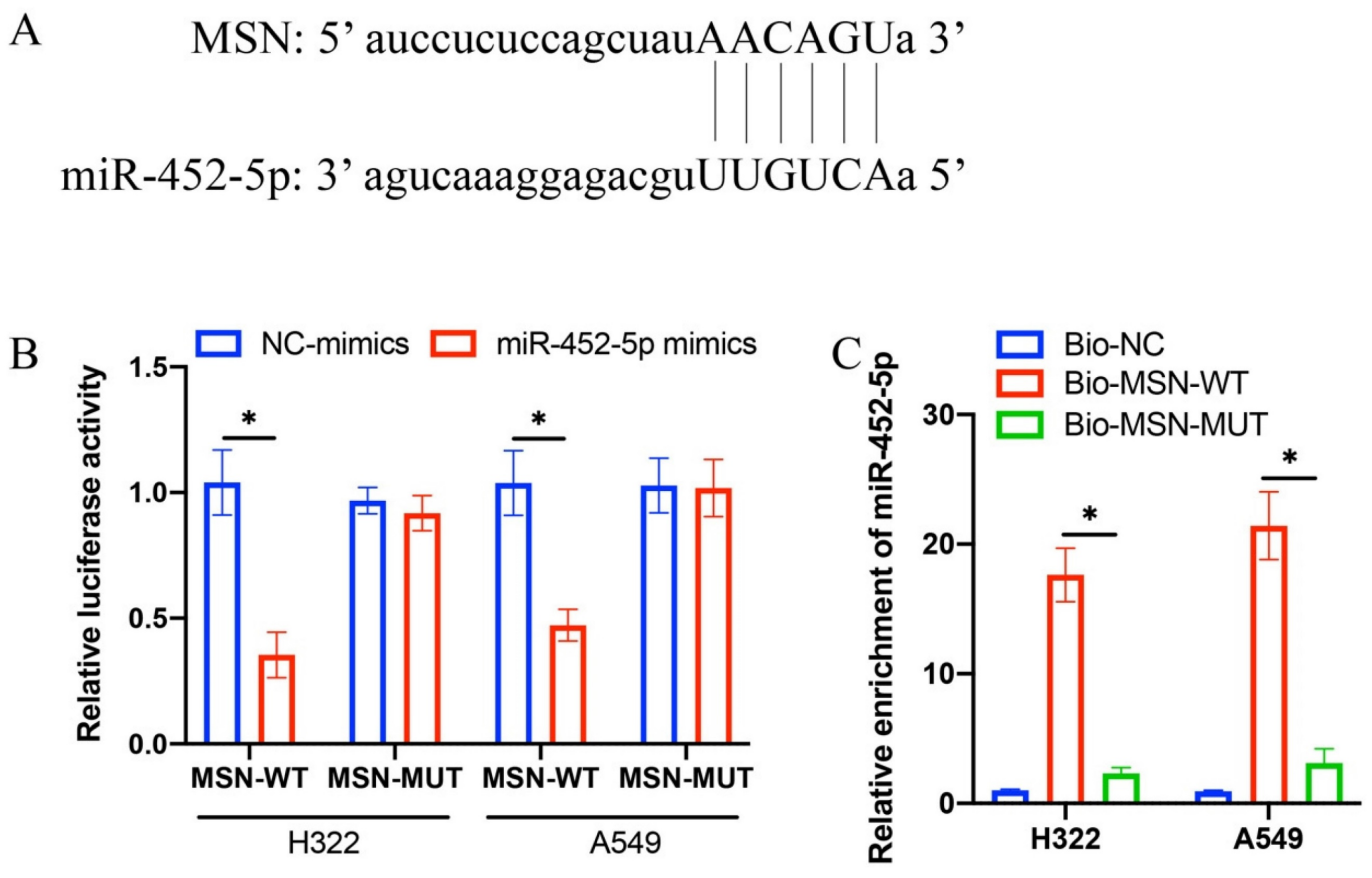
** The binding site between miR-452-5p and MSN was validated.** (A) The predication of binding site between miR-452-5p and MSN was performed through TargetScan data base; (B) Dual luciferase reporter assay was performed to validate the binding site; (C) The level of miR-452-5p was investigated. * suggests p <0.05.

**Figure 4:**
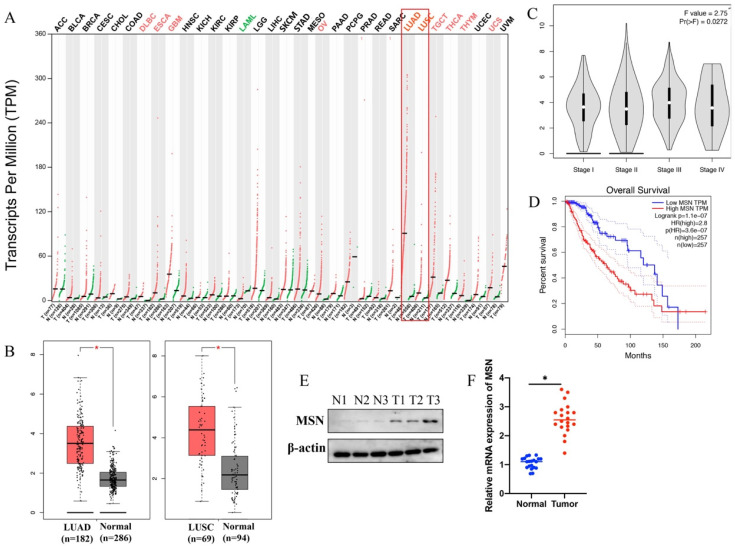
** The expression of MSN was significantly promoted in lung cancer patients, and MSN is a potential marker of lung cancer.** (A) GEPIA database was used to analyze the expression of MSN in different types of tumors; (B) Significant higher expression of MSN in LUAD and LUSC was observed; (C) Higher expression of MSN is positively correlated with advanced lung cancer; (D) Higher expression of MSN suggests poor prognosis of lung cancer patients; (E) The protein level of MSN was highly expressed in lung cancer tissues; (F) The mRNA level of MSN was highly expressed in lung cancer tissues. * suggests p <0.05.

The predication of binding site between miR-452-5p and MSN was performed through TargetScan data base (Figure 3 A). Complementary binding sequences between miR-452-5p and MSN were observed (Figure 3 A). The validation of binding site was performed through dual luciferase reporter assay. The luciferase activity of MSN-WT was greatly suppressed by miR-452-5p (Figure 3 B), and the increased level of miR-452-5p in group Bio-MSN-WT was greatly inhibited in group Bio-MSN-MUT (Figure 4 C).

### 3.4 The expression of MSN was significantly promoted in lung cancer patients, and MSN is a potential marker of lung cancer

GEPIA database was used to analyze the expression of MSN in different type tumors, the correlation between MSN expression and survival or prognosis of lung cancer. We found that the levels of MSN in different types of tumors are expressed differently (Figure 4 A). Significant higher expression of MSN in lung adenocarcinoma (LUAD) and lung squamous cell carcinoma (LUSC) was observed (Figure 4 A-B). In addition, higher expression of MSN is positively correlated with advanced lung cancer (Figure 4 C), and higher expression of MSN suggests poor prognosis (Figure 4 D). Meanwhile, the protein and mRNA levels of MSN were highly expressed in lung cancer tissues (Figure 4 E-F).

### 3.5 pcDNA-MSN significantly reversed the effects of miR-452-5p mimics on the proliferation, migration, and invasion of H322 and A549 cell lines

**Figure 5:**
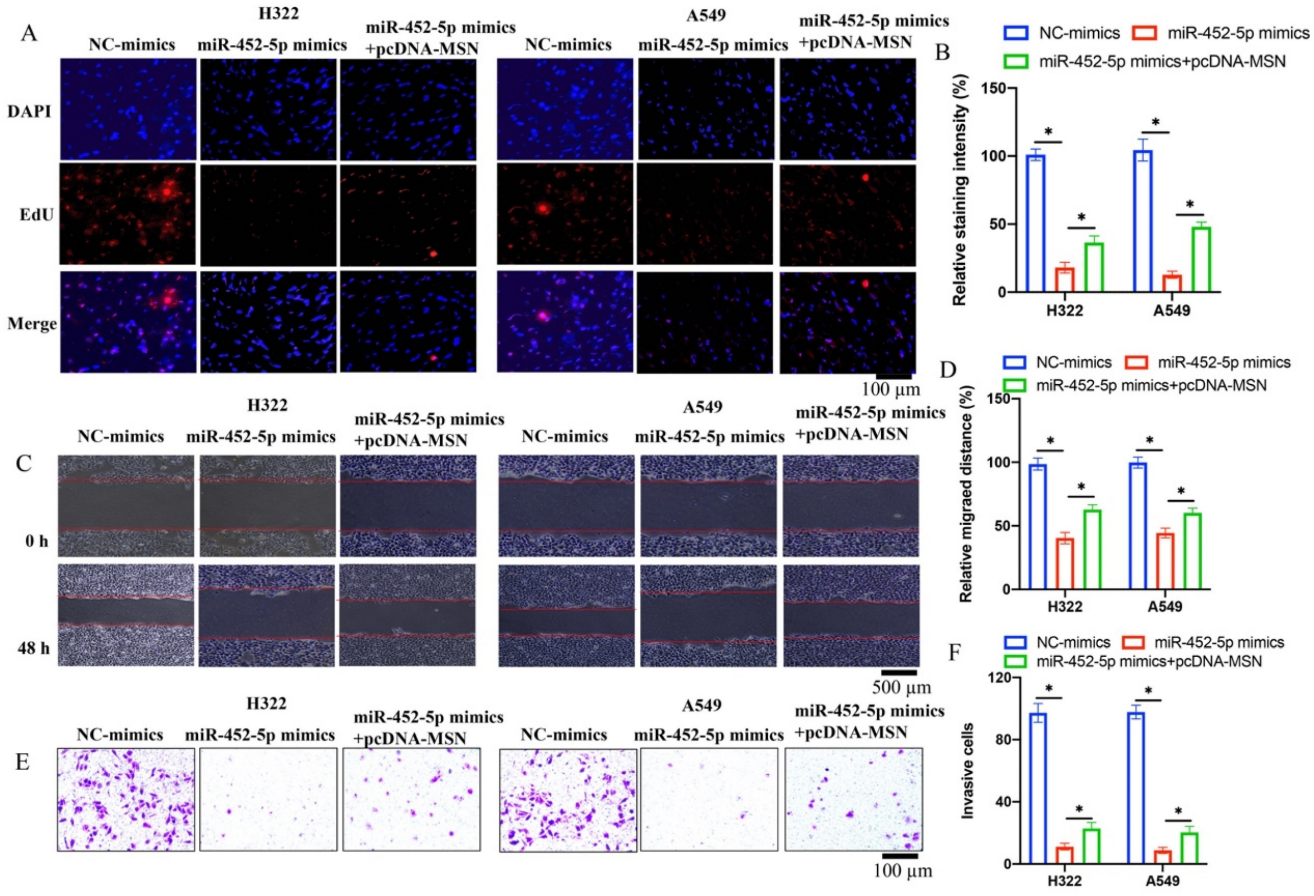
** pcDNA-MSN significantly reversed the effects of miR-452-5p mimics on the proliferation, migration, and invasion of H322 and A549 cell lines.** (A) The cell proliferation was evaluated with EdU staining method; (B) The influence of miR-452-5p on proliferation of A549 and H322 cells was reversed by pcDNA-MSN; (C) The cell migration was measured with wound healing assay; (D) The influence of miR-452-5p on migration of A549 and H322 cells was reversed by pcDNA-MSN; (E) The cell invasion was measured with Transwell assay; (F) The influence of miR-452-5p on invasion of A549 and H322 cells was reversed by pcDNA-MSN. * suggests p <0.05.

Co-transfections of miR-452-5p mimics and pcDNA-MSN in both A549 and H322 cell lines were successfully performed. We found that the suppressed proliferation (Figure 5 A-B), migration (Figure 5 C-D), and invasion (Figure 5 E-F) of H322 and A549 cell lines caused by miR-452-5p mimics were markedly reversed and promoted by pcDNA-MSN.

### 3.6 pcDNA-MSN significantly reversed the influence of miR-452-5p mimics on the EMT related proteins expression in H322 and A549 cell lines

**Figure 6:**
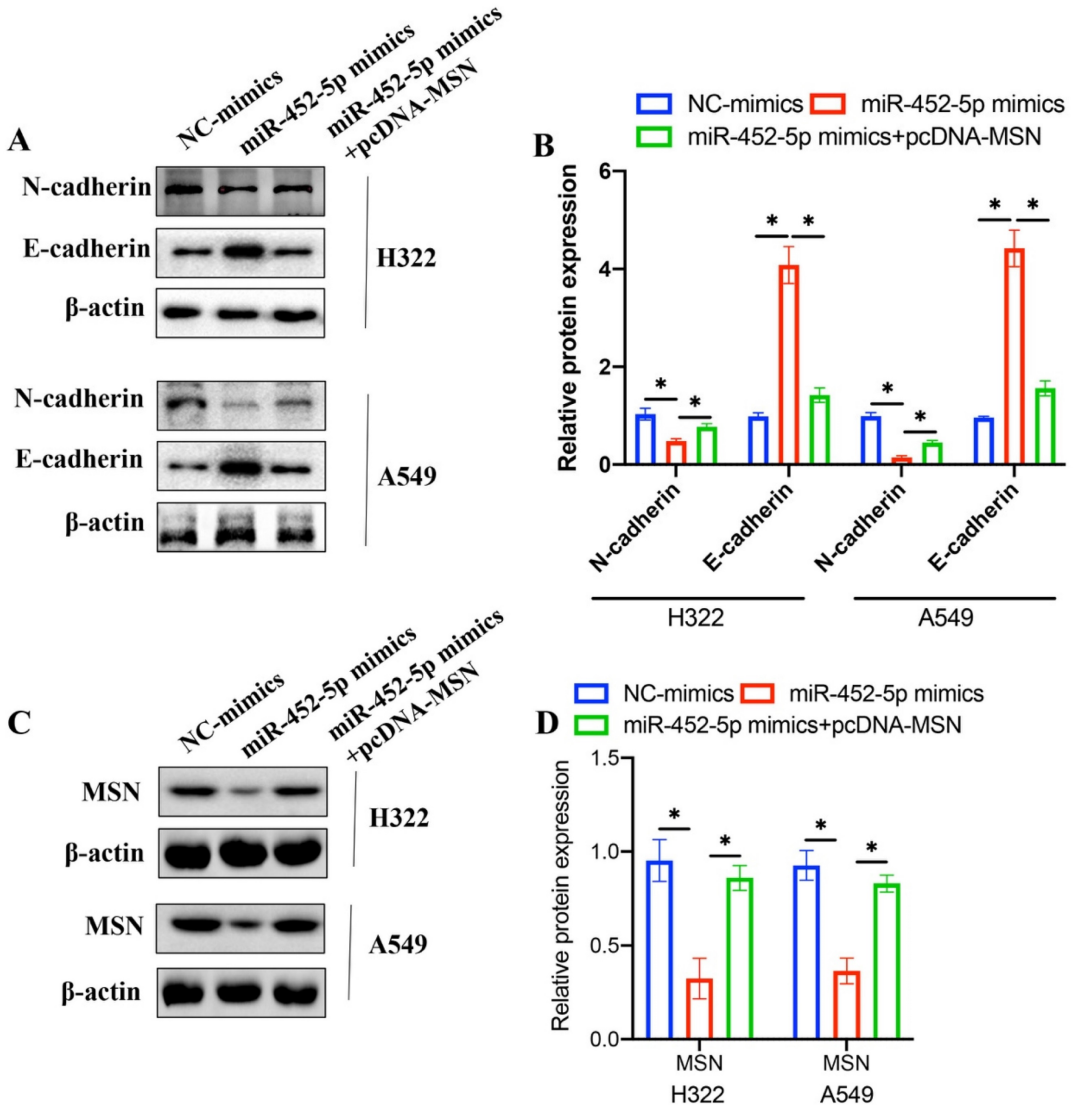
**pcDNA-MSN significantly reversed the influence of miR-452-5p mimics on the EMT related proteins expression in H322 and A549 cell lines.** (A) The expression levels of N-cadherin and E-cadherin were measured with western bolting; (B) The expression levels of N-cadherin and E-cadherin were analyzed; (C) The expression levels of MSN were measured with western bolting; (D) The expression of MSN was analyzed. * means p <0.05.

EMT process is closely linked with tumor malignancy, and the effects of miR-452-5p mimics and pcDNA-MSN on EMT related proteins were investigated. The concentration of N-cadherin was decreased, and the level of E-cadherin was increased after treatment with miR-452-5p mimics in both H322 and A549 cell lines (Figure 6 A-B). However, overexpression of MSN remarkably reversed the influence of miR-452-5p on EMT related proteins (Figure 6 A-B). These data suggest that miR-452-5p might regulate the progression of NSCLC through targeting MSN. In addition, we found that miR-452-5p greatly suppressed the protein expression of MSN, but the decreased level of MSN was promoted after transfection with pcDNA-MSN.

## 4. Discussion

Invasion and metastasis are the most important biological characteristics and signs of malignant tumors, and also the root cause of treatment failure and death of tumor patients 16. According to clinical statistics, more than 90% of lung cancer patients die of complications related to tumor invasion and metastasis 17. The invasion and metastasis of lung cancer is an extremely complex multi-gene regulation and multi-step development process, which is closely related to the biological characteristics of tumor cells, the microenvironment of organs and tissues, and the immune status of the body. We found in this research the migration and invasion of both H322 and A549 cells were markedly suppressed by miR-452-5p, but they were significantly increased by pcDNA-MSN. These data indicate that miR-452-5p might regulate NSCLC through affecting MSN. In addition, we demonstrated that miR-452-5p mimics markedly inhibited the migration, invasion, and proliferation ability of both H322 and A549 cells. However, the miR-452-5p inhibited proliferation, the effect of anti-migration and invasion may be partly through inhibiting proliferation.

miR-425-5p is located in Xq28, and it is an RNA molecule processed from the 5' end of the precursor of hsa-miR-452 18. It can target and bind multiple genes and play a role in the biological process of tumor through a variety of mechanisms 19. LncRNA-MSC-AS1 could inhibit the ovarian cancer progression by targeting miR-425-5p 20. miR-425-5p could increase tumor growth and metastasis via affecting CTNND1-mediated β-catenin pathway and EMT in colorectal cancer 21. Meanwhile, miR-452-5p could induce M2 Macrophage polarization to accelerate hepatocellular carcinoma progression via targeting TIMP3 13. miR-452-5p promoted colorectal cancer progression by regulating an ERK/MAPK positive feedback loop 18. In this research, we demonstrated that miR-452-5p suppressed the metastasis of NSCLS, indicating that the regulatory role of miR-452-5p in tumors is complicated.

EMT is considered to be one of the key processes to the metastasis and deterioration of cell carcinoma 22. At present, research has confirmed that EMT can enhance the migration and invasion ability of cells, and play a key role in the formation of tumor metastasis 23. However, due to the complex molecular mechanism, the current research in NSCLC is not very thorough. Therefore, to find the key targets of EMT in NSCLC and provide theoretical basis for further research and development of corresponding intervention and treatment methods is necessary. In this research, we found that overexpression of miR-425-5p remarkably inhibited the EMT process of NSCLC by up-regulating E-cadherin, and down-regulating N-cadherin (Figure 6 A-B).

Some studies have reported that MSN is abnormally expressed in some malignant tumor cells, and it plays an important role in the process of tumor invasion and metastasis 14, 24. MSN is an important potential target molecule of anti-tumor drugs 25. This suggests that MSN is closely related to the occurrence and development of malignant tumors. In the present study, we demonstrated that overexpression of MSN remarkably reversed the influence of miR-452-5p mimics on cell proliferation, migration, invasion, and EMT process of NSCLC cells.

In summary, we proved that miR-452-5p could modulate the cell proliferation, migration, invasion, and EMT process of H322 and A549 cell lines through targeting MSN. This research might provide a novel prevention and treatment target for NSCLC.

## Abbreviations

NSCLCL: Non-small cell lung cancer
SCLC: Small cell lung cancer
EMT: Epithelial-mesenchymal transition
miRNA: MicroRNA
MSN: Moesin
LUAD: Lung adenocarcinoma
LUSC: Lung squamous cell carcinoma
